# Direct Genetics Referral Pathway for High-Grade Serous Ovarian Cancer Patients: The “Opt-Out” Process

**DOI:** 10.1155/2019/6029097

**Published:** 2019-04-02

**Authors:** Jacob McGee, Teresa M. Peart, Norine Foley, Monique Bertrand, Michel Prefontaine, Akira Sugimoto, Helen Ettler, Stephen Welch, Karen Panabaker

**Affiliations:** ^1^Department of Obstetrics & Gynecology, London Health Sciences Centre, London, Canada; ^2^Department of Aging, Rehabilitation & Geriatric Care Program, Division of Stroke Rehabilitation and Assistive Technologies, Lawson Health Research Institute, London, Canada; ^3^Department of Pathology and Laboratory Medicine, London Health Sciences Centre, London, Canada; ^4^Department of Medical Oncology, London Health Sciences Centre, London, Canada; ^5^Department of Medical Genetics, London Health Sciences Centre, London, Canada

## Abstract

**Purpose:**

In order to meet a clinical need for better pathways to access genetic testing for ovarian cancer patients, we implemented and reviewed an opt-out referral process for genetic consultation whereby a referral is automatically sent to genetics following a pathological diagnosis of HGSC.

**Methods:**

Following implementation of the opt-out referral process, each month a list of new cases of HGSC was generated from the synoptic pathology report and forwarded directly to the Cancer Genetics clinic. Using an advanced directive, patients were automatically referred for genetic counselling two months after surgery. If the patient declined genetic counselling (opted-out) after discussion with their surgeon within the two months after surgery, the Genetic Counsellor was informed and the patient was removed from the referral process.

**Results:**

Between January 1, 2015, and December 31, 2017, 168 women were diagnosed with HGSC, of whom 167 received a referral for genetic consultation. In only one case the referral was cancelled by the surgeon, resulting in a referral rate of 99.4%. By the end of the study period, 133 women attended a genetics consultation appointment and 125 (94%) agreed to proceed with genetic testing. Among those who completed genetic testing, 15% tested positive for a* BRCA1* or* BRCA2* gene mutation. Of the women who tested positive for a* BRCA1/2 *mutation, 56% had no family history of breast or ovarian cancer.

**Conclusions:**

The opt-out referral process described in this study is s a feasible, effective, and patient-centred approach to increase access to* BRCA1/2* testing for patients with ovarian cancer.

## 1. Introduction

Epithelial ovarian carcinoma (EOC) has the highest mortality rate among gynecologic malignancies and is the fifth leading cause of cancer-related mortality in Canada [1]. High-grade serous ovarian carcinoma (HGSC), the most common subtype, presents at an advanced stage with a rather dismal 5-year survival rate ranging between 35% and 40% [2]. Women who carry a* BRCA1 or BRCA2* gene mutation are at highest risk of developing ovarian cancer, with a lifetime risk of up to 44% by 80 years of age [3]. In the past, having a family history of breast and/or ovarian cancer was the main screening criteria for referral to genetic counselling for* BRCA1/2* testing. This is not ideal given that up to 44% of women with HGSC and a documented* BRCA1 *or* BRCA2 *gene mutation do not have a family history of breast/ovarian cancer [4–7]. Therefore, we must revise how we make our decisions surrounding genetic testing for* BRCA1/2* mutations in order to more fully reach the at-risk population.

Expanding genetic testing to all women diagnosed with HGSC, regardless of ethnicity or family history, has many benefits. Firstly, it opens up new treatment options such as poly-AD-ribose polymerase inhibitors (PARP), which are most effective in women with* BRCA* gene mutations [8]. Secondly, it allows for the identification of families with a hereditary predisposition to breast and ovarian cancer. These families then have the opportunity to undergo* BRCA1/2* genetic testing and subsequent enhanced surveillance or preventative interventions. It has been estimated that testing all women diagnosed with HGSC for a* BRCA*1 or* BRCA*2 gene mutation could reduce breast and ovarian cancer in first-degree relatives by 20% and 55%, respectively [9]. Preventative prophylactic bilateral salpingo-oophorectomy following the identification of a* BRCA1 *or* BRCA2* gene mutation is associated with an 80% reduction in the risk of ovarian, fallopian tube (FT), or peritoneal cancer among* BRCA1/2* carriers and a 77% reduction in all-cause mortality [10]. This has huge long-term public health implications given the lack of effective screening modalities and the deadliness of this disease.

In response, provincial health ministries across Canada have expanded public health funding eligibility for* BRCA1/2* testing to include all women with serous ovarian cancer, including FT and peritoneal cancer. Despite this expansion of funding for BRCA mutation testing in Ontario in 2006, the subsequent ten years saw less than 10% of patients diagnosed with HGSC completing genetic consultation [11]. This extremely low number of patients completing genetic consultation and testing can largely be attributed to limitations in the systems through which ovarian cancer patients are referred for testing [12].

Review of the Ontario Ministry of Health data associated with expanded coverage for* BRCA1/2* testing has revealed important predictors of referral for genetic counselling such as: race (White), parity (>0 children), histology (serous histology), tumor site (FT), and family history of breast and/or ovarian cancer [11, 13]. In particular, women who discussed a genetic risk of cancer with their physician(s) within the first three visits were more likely to receive a referral for genetic consultation [14]. In contrast, negative predictors for referral included increasing age and lack of family history of breast or ovarian cancer [11, 14, 15]. Cancers identified at later stages of the disease process have also been associated with lower referral rates. Additionally, genetic consultation referral opportunities are often missed when multiple care providers are involved if no one provider takes responsibility for referral [11].

Clearly, improvement in the referral process is required to enable increased access to genetic consultation for women with HGSC. In response, the London Regional Cancer Program (London, Ontario, Canada) changed its referral process from an “opt-in” to an “opt-out” approach in 2015. Using this approach, all new cases of HGSC are now directly referred for genetic counselling using an advanced medical directive. If the patient declines genetic counselling during their postsurgery pathology review meeting with their surgeon (opting out), their surgeon is required to inform genetics. The primary objective of this study is to describe the outcome of the opt-out referral process among women newly diagnosed with HGSC, specifically: (1) the numbers of women who declined or accepted direct referral through the opt-out referral process and (2) the results of genetic testing (i.e.,* BRCA1/2* positive or negative) among women who consented to testing.

## 2. Methods

### 2.1. Study Setting and Design

A prospective observational cohort study was conducted between January 2015 and December 2017, to evaluate a quality improvement project initiated by the Division of Gynecologic Oncology at the London Regional Cancer Program and the Cancer Genetics clinic at the London Health Sciences Centre (LHSC). This quality improvement project was undertaken in accordance with Western Research guidelines. All women ≥18 years of age who had surgery at a tertiary gynecologic oncology referral center, newly diagnosed with a first-occurrence of a pathologically confirmed HGSC (including peritoneal and FT carcinomas), were eligible for direct referral to the Cancer Genetics clinic for a genetic consultation. The referral process was not initiated for women who were treated outside the South West Local Health Integration Network (LHIN). During the course of the study period, the molecular genetics laboratory at LHSC transitioned from offering only BRCA1/2 gene analysis to NGS panel testing (launched March 2016). Additionally, the number of genes included on the panel increased during the study period (from a 16-gene panel to a 37-gene panel between March 2016 and December 2017).

### 2.2. Genetic Consultation Referral Process

Monthly, a list of all new HGSC patients identified through an automated search from synoptic pathology reports was generated from patient electronic medical records and then forwarded directly to the Cancer Genetics clinic. The diagnosis of HGSC prompted direct referral for genetic counselling via an advanced directive, the use of which was agreed upon by the four gynecologic surgical oncologists at LHSC. Two months after the surgery date, a letter was sent to the patient, acknowledging their Cancer Genetics referral by their surgeon. The letter also included an appointment date for genetic consultation, the purpose of which was to discuss their eligibility for genetic testing. Patients also received a family history screening questionnaire that was to be completed and returned to the Cancer Genetics clinic prior to their consultation.

**Figure 1:**
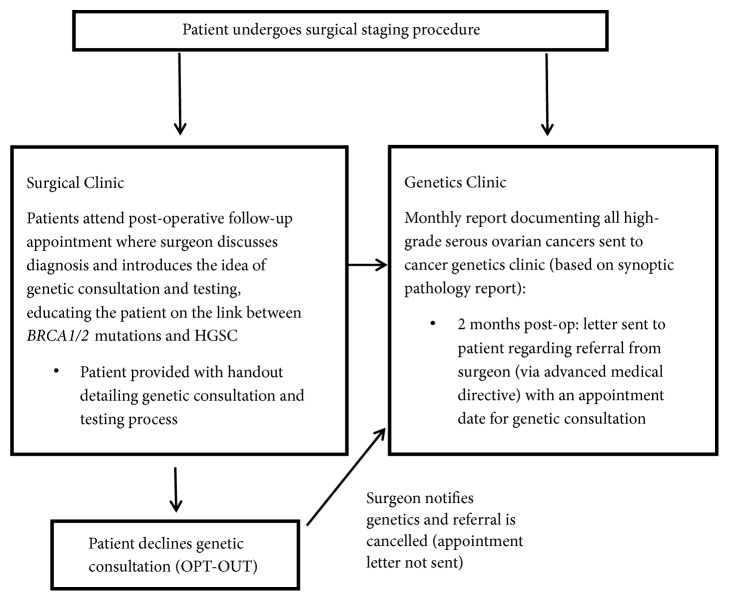
Opt-out pathway for* BRCA* testing in ovarian cancer patients.

Patient pathology reports were reviewed during the first postoperative appointment with the surgeon following cytoreduction. During this appointment, patients were (1) briefly educated on the association between HGSC and* BRCA1/2* gene mutations by their surgeon and/or nurse, (2) provided with a handout detailing the purpose of genetic counselling and the testing process (Appendix A), and (3) informed of the opt-out referral process for genetic consultation. Should the patient decline genetic consultation at the time of their pathology review, thereby opting out of the process, their surgeon informed the Cancer Genetics clinic, and they were removed from the referral process (Figure 1).

### 2.3. Data Collection and Processing

Data collection was conducted by chart review and included age, cancer stage, and family history of cancer. The main study outcomes were those associated with the referral process, genetic consultation, and results of genetic testing and are presented as dichotomous variables and summarized as proportions. Referral process outcomes included the proportion of patients who (1) opted-out of the referral process for genetic counselling appointments and (2) held their appointment for genetic counselling. Genetic consultation outcome was determined as the proportion of patients who agreed to genetic testing. The efficiency of the referral process was determined as the median time from when the genetics referral was sent to when the initial genetic consultation appointment was completed. Genetic testing outcomes were reported for those who had agreed to and completed genetic counselling during the study period. Genetic testing outcomes included the proportion of women who tested positive for* BRCA1* and/or* BRCA2* gene mutations. Genetic testing results associated with variants of uncertain significance (VUS) as well as pathogenic gene mutations other than* BRCA1/2* identified through next generation sequencing (NGS) were also recorded.

## 3. Results

During the study period, there were 168 women newly diagnosed with HGSC who were eligible for genetic consultation, of whom, 99.4% accepted genetic consultation. Patient characteristics are presented in Table 1.

Only one woman opted-out of the referral process and had her appointment cancelled by their surgeon (Table 2), resulting in a referral rate of 99.4%. Of the 167 patients with booked appointments, 34 patients (20.4%) did not attend their genetic consultation appointment. Reasons for appointments that were not held included: patient declined appointment (n=10; 29.4%); patient accepted the appointment but later cancelled (n=14; 41.2%); patient died before appointment could be held (n=8; 23.5%). By the end of the study period, 133 patients held their genetic consultation appointment, of which 125 (94.0%) women decided to proceed with genetic testing and 8 women declined testing (Table 3). The median time from genetic referral to consultation was 3 months (range 1-8 months).

Of the 123 women whose test results were available at the time of this review, 14.6% tested positive for either a* BRCA1* or* BRCA2 *gene mutation. Variants of uncertain significance (VUS) were detected in 9 patients, and pathogenic mutations in non-*BRCA* genes were found in 8 patients (Table 4). Among women who tested positive for either a* BRCA*1 or* BRCA*2 gene mutation, 56% had no previous family history of breast or ovarian cancer (Table 5).

## 4. Discussion

The opt-out referral strategy, in which all eligible women diagnosed with HGSC were directly provided with an appointment for genetic counselling, resulted in significantly more patients undergoing genetic consultation and subsequent testing compared with historical levels [11]. Of the patients who received genetic counselling, 94% underwent testing, a result significantly higher than that observed in other regional referral systems like Princess Margaret Hospital (Toronto, Ontario Canada) and the Juravinski Cancer Centre (Hamilton, Ontario, Canada). A review of the referral process at the Juravinski Cancer Centre, which relies on referral by physician, found that despite universal eligibility for* BRCA* genetic testing for all women diagnosed with HGSC, only 32% of eligible patients were referred for genetic counselling [14], compared with 99% in the present study. Although it has been noted that family history is a poor triage criteria for* BRCA* testing in ovarian cancer patients, physicians remain stringent in their referral patterns for genetic consultation, resulting in lower referrals [12, 13]. Since our opt-out referral process eliminates bias and bypasses the need for direct involvement by the physician in the referral process, we were successful in ensuring that all eligible women were provided with the opportunity for genetic consultation and testing.

Currently, funding guidelines have been updated to outline who should be referred for genetic consultation but the systems through which cancer patients are able to access this testing have yet to be standardized. The opt-out referral system we describe in this study is a feasible way to ensure that all eligible patients are referred for genetic consultation and testing and can be implemented wherever synoptic pathology reporting is utilized. In the province of Ontario, all ovarian cancers are referred to one of five tertiary care hospitals, all of which use synoptic reporting. Adoption of an opt-out referral system across the province of Ontario for genetic consultation would greatly increase referral rates and eventual genetic testing for women with HGSC.

The two-month lag time after surgery used in this study allowed physicians to see patients postoperatively, discuss the diagnosis and future plans for treatment, and introduce the idea of genetic counselling. Women who are able to discuss genetic consultation with their physician within the first three visits following their surgery were more likely to have the testing done [14]. Accordingly, Demsky et al. (2013) documented that 99% of women who were seen for genetic consultation pursued genetic testing. Indeed, we observed that, of those women who received genetic counselling, 94% of them pursued genetic testing [15].

As other centres adopt the opt-out process described in this study, there will be some hurdles that need to be overcome and other considerations to be made. With a high mortality rate disease like HGSC, an important factor to consider is time from initial referral to completion of genetic consultation appointment. Despite a rather efficient median time from referral to consultation appointment of 3 months, 8 genetic consultation appointments were cancelled because the patient died prior to the appointment date. Furthermore, 10 patients declined the appointment when contacted, and 14 others failed to attend their appointment. A potential explanation for this is the morbidity associated with the cumulative effects of surgery and chemotherapy, as well as appointment fatigue as treatment progresses; however a thorough assessment why these appointments did not occur is beyond the scope of this study. In this regard, mainstreaming, another option for increasing genetic referral rates, may provide a more streamlined process and allow for quicker access to testing [12]. The main difference between the mainstreaming pathway and the opt-out pathway described in this study is that mainstreaming eliminates the need for a genetic consultation appointment by training members of the cancer care team to obtain the consent and samples for testing. The strength of the opt-out pathway over this method, however, is that referrals are automatic for all HGSC and do not require direct involvement of the surgeon, apart from a brief discussion of a possible association between HGSC and a genetic mutation, in the referral process.

The increased uptake of genetic counselling and subsequent genetic testing observed in this study using an opt-out referral strategy has many benefits for the patient as well as the patient's family. The presence of a* BRCA* mutation may provide a therapeutic benefit for the patient who will now be eligible to be treated with PARP inhibitors, which have been shown to prolong progression-free survival in patients with platinum-sensitive disease [16–18]. Additionally, knowledge of* BRCA* status will allow the patient's family to be tested, and where results are positive, these individuals then have the opportunity to undergo risk-reducing medical and surgical interventions. Where patients decline testing for their direct benefit, DNA banking may be offered so their family has the option to have the DNA tested at a later date. Where DNA banking is considered, it is important that the patient's and family's preference for family members to contact, and who from the medical team should contact them, are documented, as well as the timing of results disclosure [19]. Given the deadly nature of this disease and the lack of efficient screening strategies for early detection, opportunities for true prevention must be embraced.

Indeed, among the patients who completed the genetic counselling and genetic testing process, 14.6% tested positive for either a* BRCA1* or* BRCA2 *gene mutation, a percentage similar to other documented reports [20]. Of the patients who tested positive for* BRCA1/2* mutations, 56% had no previous family history of breast or ovarian cancer. This has important implications for the families of these patients who now may consider undergoing genetic testing as well. Implementation of strategies, such as this opt-out process, to increase genetic referral rates for eligible patients across the province of Ontario has long-term economic and public health benefits as it allows for identification of the at-risk population before they develop disease.

The opt-out pathway for genetic testing we describe in this study could be implemented at other centres across the country or even internationally, wherever synoptic pathology reporting is used. We have demonstrated it to be an effective pathway to increase access to genetic testing for ovarian cancer patients. This pathway could potentially be applied to other cancer sites, like triple negative breast cancer, where genetic consultation is recommended.

## Figures and Tables

**Table 1:** Patient characteristics.

Characteristics	N =168
Age, years	
Mean (±SD)	67 (±10)
Median (IQR)	68 (40 -86 )
Age (years)	
< 44 years	2
45–64	63
65–80	91
> 80	12
Stage	
1-2	22
3-4	140
Unknown	6
Family history*∗*	
History of BRCA-associated cancers	30
History of Lynch-associated cancers	13
History of both BRCA and Lynch-associated cancers	16
No cancer history	53
Unknown/other history	56

^*∗*^Family history based on at least one first-degree relative or two second-degree relatives with a cancer diagnosis of known origin.

**Table 2:** Opt-out pathway referral outcomes*∗*.

*Referral outcome (n=168)*	N (%)
Opt-out of referral process (cancelled by surgeon)	1 (0.6%)

Opt-in to referral process	167 (99.4%)

*Appointments held (n=167 )*	

Completed or test results pending	133 (79.6 %)

*Appointments not held (n=167 )*	34 (20.4 %)

Appointment declined by patient	10 (29.4%)

Patient died before appointment date	8 (23.5%)

Patient cancelled appointment	14 (41.2%)

Patient moved out of hospital network	2 (5.9%)

^*∗*^Referral outcomes are based on 168 women with a new diagnosis of high-grade serous cancer with booked appointments for genetic consultation via the opt-out referral pathway during the study period of January 2015 to December 2017.

**Table 3:** Genetic consultation outcomes*∗*.

Genetic consultation outcomes (N=133)	N (79.2%)
*Appointment completed*	133 (100 %)
Patient declined genetic testing	8 (6.0%)
Patient decided to proceed with genetic testing	125 (94.0%)

^*∗*^Genetic consultation outcomes are based on the 133 women with a new diagnosis of HGSC who had held their booked appointment for genetic consultation between the study period of January 2015 to December 2017.

**Table 4:** Genetic testing outcomes*∗*.

Genetic testing results (N=125 )	N (75.0%)
*Genetic testing completed*	N=123 (98.4%)

Positive *BRCA 1* or *BRCA 2*	18 (14.6 %)

Negative *BRCA 1* or *BRCA 2*	17 (13.8%)

Negative NGS panel (including *BRCA1/2*)	52 (42.3 %)

Positive non-BRCA gene on NGS panel*∗∗*	8 (6.5 %)

VUS – *BRCA1* or *BRCA2*	9 (7.3 %)

VUS other*∗∗∗*	21 (17.1 %)

*Genetic testing pending*	2 (1.6 %)

NGS: next generation sequencing; VUS: variant of uncertain significance.

^*∗*^125 patients had undergone genetic testing during January 2015–December 2017. Results of the genetic tests were known for 123 patients, while results were pending for 2 patients.

^*∗∗*^Non-BRCA pathogenic variants detected: *BRIP1*, *MUTYH* heterozygote, *PALB2*, *RAD51C*, and *TP53*(mos).

^*∗∗∗*^Some patients had more than one VUS in multiple genes.

**Table 5:** Family history risk categories for patients with mutations in *BRCA1/2*.

BRCA positive (N=18)	N (14.6%)
Average Risk	10 (55.6%)
Moderate Risk	5 (27.8%)
High Risk	3 (16.6%)

Average Risk = no first- or second-degree relatives with breast or ovarian cancer.

Moderate Risk = one first-degree relative with breast or ovarian cancer or two first or second-degree relatives with pancreatic or prostate cancer.

High Risk = at least one first-degree and second-degree relative diagnosed with breast or ovarian cancer on the same side of the family or three or more first- or second-degree relatives with breast, ovarian, prostate, or pancreatic cancer.

## Data Availability

The compiled data used to support the findings of this study are included within the article. Additional data is not available due to maintain privacy and confidentiality of study subjects.

## Appendix

### A. Genetic Consultation/Testing Handout

#### A.1. Cancer Genetics, Genetic Testing, and DNA Banking

What is Cancer Genetics?

Sometimes it can seem that cancers “run in the family”. Cancer Genetics can identify a specific gene mutation (a change in a gene that prevents it from working properly) that may have been passed down from one generation to the next. This can sometimes increase the risk of cancer in some people. Our Cancer Genetics clinic offers education, genetic counselling, and sometimes genetic testing for patients who suspect that there might be a genetic link in their family.

Why am I being referred to the Cancer Genetics clinic?

You are being referred to the Cancer Genetics clinic because you have been diagnosed with a certain type of ovarian cancer known as “serous” ovarian cancer. A woman with a diagnosis of serous ovarian cancer, at any age, now qualifies to have genetic testing. When your referral is made to the Cancer Genetics clinic, you will receive a letter directly from the Cancer Genetics clinic with your appointment date and time, and a questionnaire for you to complete. The letter will provide a phone number for you to call if you have any questions at all. At your appointment, the Genetic Counsellor will discuss genetic testing and what the test results might mean for you and your family. All of these services are covered by your Ontario Health plan.

What could the benefits of genetic testing be for me?

Some patients find it helpful to understand why they got their cancer. Finding out that you were born with an abnormal gene that has contributed to the development of your ovarian cancer can answer many questions. There are always new cancer treatments being tested and certain treatments (e.g., specific chemotherapy drugs) may work better on cancers that are found in people who were born with gene changes. By doing genetic testing, we may learn which treatments might work best for you in the future.

What could the benefits of genetic testing be for my family?

If we know that there is an inherited gene change in the family, your children or other relatives may also decide to have genetic testing to see if they have a higher risk of developing cancer. Knowing that you have an inherited gene change does not necessarily mean that your children will have it too. By understanding the benefits of genetic testing, your family can have earlier and regular monitoring or surgery to try to reduce their risk. It may also help to prevent them from getting certain types of cancers.

What are the risks of genetic testing?

Genetic testing does not have any physical or medical risks. The test is a simple blood test. Results from the test may be stressful for some patients. Some find it upsetting to find out that they have a gene change that they might have passed down to their children. Some patients also find it difficult to tell family members.

Do I have a choice about meeting with a Genetic Counsellor in the Cancer Genetics clinic?

Yes. You are going to get a letter directly from the Cancer Genetics clinic with your appointment date and time and a questionnaire for you to complete. The letter will provide a phone number for you to call if you have any questions at all before the appointment, or if you wish to speak with a Genetic Counsellor before deciding if you want an appointment. You can say “no” at any time if you decide that you do not want to go any further. You can also just meet with a Genetic Counsellor to learn more about what hereditary ovarian cancer is, and what it means to have genetic testing. At the end of your appointment, you have the choice to say “no” to genetic testing or you may say “yes” and have a blood sample drawn.* It is your choice as the patient*.

What if I do not want to meet with anyone at this time but might want testing in the future?

If you are not interested in meeting with a Genetic Counsellor at this time, you could still have a blood test done. The DNA from your blood sample can be kept in our laboratory for possible use in the future by your family. This option is called DNA banking. Genetic testing is not done on a stored DNA sample. The patient who provided the sample, or their legal next of kin, must meet with a Genetic Counsellor and give informed consent before any genetic testing can be performed. If you would only like to have a blood test for DNA banking at this time, please contact the Cancer Genetics clinic at 519-685-8727.
